# The impact of pastoralist mobility on tuberculosis control in Ethiopia: a systematic review and meta-synthesis

**DOI:** 10.1186/s40249-019-0583-z

**Published:** 2019-09-02

**Authors:** Faisal Nooh, Lisa Crump, Abdiwahab Hashi, Rea Tschopp, Esther Schelling, Klaus Reither, Jan Hattendorf, Seid M. Ali, Brigit Obrist, Jürg Utzinger, Jakob Zinsstag

**Affiliations:** 1 Swiss Tropical and Public Health Institute, P.O. Box, CH-4002, Basel, Switzerland; 2 University of Basel, P.O. Box, CH-4003, Basel, Switzerland; 3 Jigjiga University, P.O. Box, 1020, Jigjiga, Ethiopia; 4 Armauer Hansen Research Institute, P.O. Box, 1005, Addis Ababa, Ethiopia

**Keywords:** Directly observed treatment, short-course (DOTS), Effectiveness, Equity, Ethiopia, Meta-ethnographic method, Pastoralist, Systematic review, Tuberculosis

## Abstract

**Background:**

Directly observed treatment, short-course (DOTS) is the current mainstay to control tuberculosis (TB) worldwide. Context-specific adaptations of DOTS have impending implications in the fight against TB. In Ethiopia, there is a national TB control programme with the goal to eliminate TB, but uneven distribution across lifestyle gradients remains a challenge. Notably, the mobile pastoralist communities in the country are disproportionately left uncovered. The aim of this study was to summarize the evidence base from published literature to guide TB control strategy for mobile pastoralist communities in Ethiopia.

**Main text:**

We followed the Preferred Reporting Items for Systematic Reviews and Meta-Analyses (PRISMA) guidelines and systematically reviewed articles in seven electronic databases: Excerptra Medical Database, African Journal Online, PubMed, Google Scholar, Centre for Agriculture and Bioscience International Direct, Cochrane Library and Web of Science. The databases were searched from inception to December 31, 2018, with no language restriction.

We screened 692 items of which 19 met our inclusion criteria. Using a meta-ethnographic method, we identified six themes: (i) pastoralism in Ethiopia; (ii) pastoralists’ livelihood profile; (iii) pastoralists’ service utilisation; (iv) pastoralists’ knowledge and awareness on TB control services; (v) challenges of TB control in pastoral settings; and (vi) equity disparities affecting pastoralists. Our interpretation triangulates the results across all included studies and shows that TB control activities observed in pastoralist regions of Ethiopia are far fewer than elsewhere in the country.

**Conclusions:**

This systematic review and meta-synthesis shows that TB control in Ethiopia does not align well with the pastoralist lifestyle. Inaccessibility and lack of acceptability of TB care are the key bottlenecks to pastoralist TB service provision. Targeting these two parameters holds promise to enhance effectiveness of an intervention.

**Electronic supplementary material:**

The online version of this article (10.1186/s40249-019-0583-z) contains supplementary material, which is available to authorized users.

## Multilingual abstracts

Please see Additional file 1 for translations of the abstract into the five official working languages of the United Nations.

## Background

Tuberculosis (TB) is one of the oldest recorded diseases [1] and remains a global health challenge despite relentless efforts to conquer it. In 2017, TB was among the top ten causes of death worldwide and the World Health Organization (WHO) reported that TB caused more than 10 million new cases and 1.6 million deaths [2]. TB is also a leading cause of death in people in the most economically productive age groups.

Despite a decreasing trend in TB mortality (12% reduction between 2013 and 2017 [2]) and strong political commitment to control it [3], the disease remains a serious public health problem in Ethiopia. According to WHO data in 2018, Ethiopia was among the top 14 countries in terms of burden due to TB, TB-HIV co-infection and multi-drug resistant (MDR) TB [2]. In 2017, nearly 28 600 people died from TB in Ethiopia and the total incidence rate for all forms of TB was estimated at 172 (range 121–232) per 100 000 population [2]. The first population-based nationwide survey in 2010/2011 recorded a point prevalence rate of 240 (range 182–298) per 100 000 population, with pastoral areas bearing the highest burden [4].

Although the national TB control programme brought about substantial improvements in plans to eliminate TB [5], an uneven distribution, with unfair and avoidable differences in TB control services across lifestyle gradients (gap in equity-effectiveness), remains. Mobile pastoralist communities are disproportionately left uncovered and TB control in pastoral settings remains poor [5]. Identifying the challenges affecting the TB control programme in the pastoral community is imperative to improve the situation. To better understand the problem and develop foundations for context-specific TB control programmes for pastoralists, we reviewed the available literature to evaluate the current status of TB control in Ethiopian pastoralists and to analyse its impact. We applied an equity-effectiveness model developed by Zinsstag and colleagues [6] based on an analytical framework presented by Obrist et al. [7].

## Methods

The preparation and reporting of this systematic review followed the Preferred Reporting Items for Systematic Reviews and Meta-analysis (PRISMA) guidelines [8], adhering to a study protocol developed beforehand. Our review was complemented with a qualitative approach, using Noblit and Hare’s meta-ethnographic method [9]. Of note, meta-ethnography is used to summarise results of multiple qualitative studies. Although some scholars limit its applications to ethnographic studies only, meta-ethnography is broadly applicable for all kinds of qualitative studies, according to Noblit and Hare.

The impact of the results on equitable TB control in Ethiopia was assessed following the equity-effectiveness model, as described elsewhere [6]. In brief, effectiveness of interventions at community level substantially decreases along a pipeline of parameters that measure access to, and quality of, care. Access parameters include availability, accessibility, acceptability, affordability and adequacy of service. Quality of care parameters are targeted accuracy, compliance and adherence. The probabilistic product of these parameters provides an estimate of community-effectiveness of a particular service.

### Study characteristics

The systematic review analysed both original studies and grey literature according to the following criteria.

#### Population

The populations of interest were pastoralists in Ethiopia. Of note, a considerable part of the population in Ethiopia depend on livestock as their main livelihood asset [10].

#### Intervention

Studies and reports addressing the access components (i.e. accessibility, availability, affordability, acceptability and adequacy) [7] of TB control services, in particular, those considering the challenges of TB control and different models of adapting directly observed treatment, short-course (DOTS) strategy in pastoralist settings, were included.

#### Comparator

Studies reported from Ethiopian regions where the majority of the population have a sedentary lifestyle were the comparator.

#### Outcome

The primary outcome was community-effectiveness of TB control services across livelihood styles. Community-effectiveness is a measure of the efficacy of interventions at community level and is a composite of different parameters, such as access, targeted accuracy, users’ adherence and compliance of providers [6].

### Bibliographic databases included

Potentially eligible literature was sought from readily available electronic databases; namely, Excerptra Medical Database, African Journal Online (AJOL), PubMed, Centre for Agriculture and Bioscience International (CABI) Direct, Cochrane Library and Web of Science. We searched the databases from inception to December 31, 2018 without language restriction.

### Search strategy

The electronic literature search of studies and reports was conducted using keywords developed according to the population, intervention, comparator and outcome (PICO) framework and word combination strings developed with Emtree and MeSH indexing and the help of experts. Two strings of word combinations were employed to identify relevant research: (i) tuberculosis OR tb OR “agricultural worker’s disease” OR dot* OR “directly observed therapy” OR “Directly observed treatment”; and (ii) pastoralis* OR nomad* OR transhuman* OR agropastoral*.

### Selection of studies

No restriction rules were applied during the search. Duplicates were removed. Titles and/or abstracts of identified studies were screened for relevance. Full text items were evaluated for inclusion based on eligibility criteria. The search was supplemented with hand searching of the reference lists of relevant items. Grey literature, such as government reports, was identified using Google Scholar. Identified items were transferred to the bibliographic software, Endnote™ X8 (Clarivate Analytics; Philadelphia, PA, USA), with full text documents imported to Atlas.ti™ 7 (ATLAS.ti Scientific Software Development GmbH; Berlin, Germany).

### Data extraction

We extracted data from quantitative studies retained for analysis to a Microsoft® Excel 2010 (Microsoft Corp; Washington, USA) data collection form. Data included: study characteristics, participant characteristics and outcomes. We used Atlas.ti™ 7 (https://atlasti.com/) interpretive coding to extract qualitative data from included documents.

### Risk of bias in individual studies

Two independent reviewers assessed the risk of bias in all studies included for quantitative studies. Two additional experienced reviewers independently assessed the risk of bias in samples of the included studies. Disagreement between reviewers was discussed until agreement was found. Although the risk of bias in qualitative studies is high and subject of debate [11], special emphasis was given to systematically retrieve, analyse and interpret the qualitative information.

### Data synthesis

For the quantitative data, descriptive findings were summarised in tables and graphs. Data from qualitative studies were synthesised by thematic analysis using a meta-ethnography approach [9]. Findings from primary studies were coded and categorised in groups using Atlas.ti™ 7. Themes were identified from the categories. For hermeneutic interpretation, both quantitative and qualitative results were combined using convergent data analysis [12].

## Results

### Description of search results

**Fig. 1:**
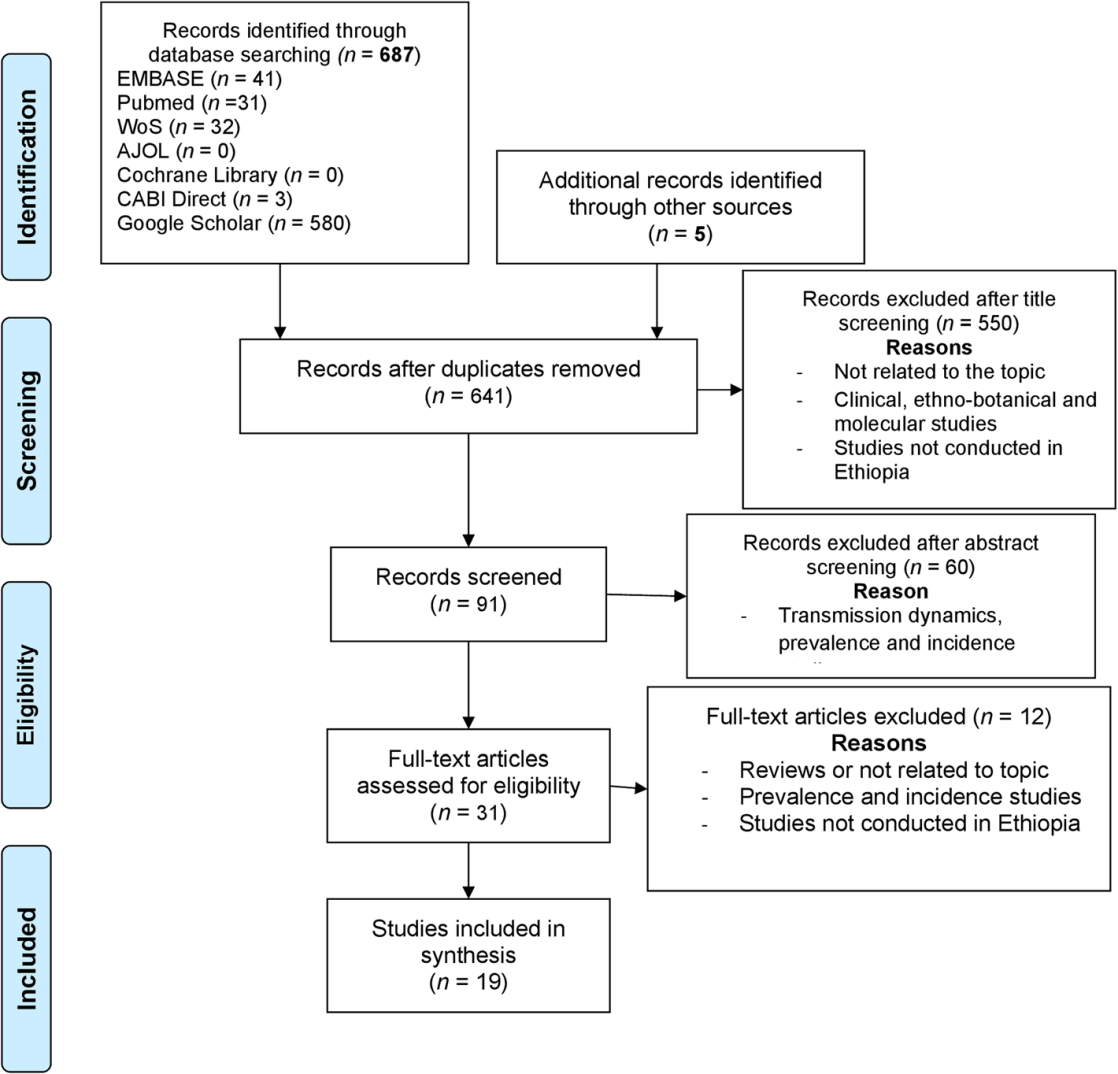
PRISMA flow chart for selection of included items. *Adapted from*: Moher D, Liberati A, Tetzlaff J, Altman DG, The PRISMA Group (2009). Preferred Reporting Items for Systematic Reviews and Meta-Analyses: The PRISMA Statement. PLoS Med 6(7): e1000097. doi:10.1371/journal.pmed1000097

The bibliographic database search identified 687 items. In total, 84% of the identified items were from Google Scholar, and most of the included studies were derived from PubMed. A manual search of references yielded another five items. A total of 31 items were retained after screening for duplications and relevance. Further analysis based on eligibility criteria produced 19 items for final inclusion and analysis (Fig. 1).

### Description of studies

Of the 19 included studies, 13 were conducted in the Somali and Afar regions, two were conducted in two pastoralist zones of the predominantly non-pastoralist Oromia region and the remaining four consisted of national level data. Sixteen were journal articles and three were government reports.

Four studies [13–16] focused on pastoralists’ knowledge, attitudes and perceptions of TB, while four studies [17–20] used time series data to analyse trends in TB control with particular consideration for treatment outcome. Two studies described and evaluated modified forms of TB treatment strategies [21, 22]. The remaining nine studies addressed challenges pastoralists face along the TB care continuum [23–31].

Some of the studies were judged to be of low quality. These contributed little to the meta-synthesis, thus limiting their influence. Further details on study characteristics are presented in Additional file 2.

### Description of themes

The synthesis identified six themes.

#### Pastoralism in Ethiopia

Pastoralists are mobile people whose livelihood depends on livestock. Although pastoralists use mobility to respond quickly to fluctuations in natural resource availability, they are experiencing an evident gradual shift from nomadic herding towards a sedentary way of life driven by multiple factors, including increased sedentary population, environmental stresses and changes in policies and practices, such as restricting access to land and water [29]. Gele [24] provides an account of the migratory lifestyle of selected pastoralists in Somali Regional State (SRS), identifying two types of pastoralism, nomadism and agro-pastoralism, which are characterised by differential access to health care. The study found that the migratory pattern in the region is predictable and suggested a “strategic villages” approach for TB service delivery. Tyler-Smith and colleagues [21] report on a “TB village” approach, similar to that suggested by the previous study [24], where food, shelter and utilities are provided and a self-administered treatment (SAT) strategy is applied. Similarly, Khogalia [22] conducted a cohort study using SAT strategy in a similar setting but did not provide shelter and food. Though the former study found higher treatment success rate and lower default rate than the later, both of these intervention studies reported higher default rates compared to a 10-year trend analysis [18] from health facilities applying DOTS in the same region.

#### Pastoralists’ livelihood profile

Access to livelihood assets, including local knowledge, skills, social connections and livestock, has a large influence on health seeking behaviour [7]. TB is well known to pastoralists, but the biomedical concept of the cause of TB is limited. A majority of the pastoralists perceive the cause to be insufficient food intake [13, 15, 16], cold air [13, 14, 16], witchcraft [14, 15] or smoking [14, 16].

Livestock is the mainstay of pastoralist livelihood. For financial liquidity, for instance, cash for nutritional supplements and accommodation during the intensive phase of treatment, they might sell livestock [24].

#### Pastoralists’ service utilization

Pastoralists make use of different service utilization strategies. Most commonly, they initially contact traditional and religious healing practitioners or informal conventional health care practitioners in local pharmacies and private clinics [15, 24, 26]. In advanced stages of a disease, they contact public health centres and hospitals [24]. Availability of health extension workers helps pastoralists find suitable and appropriate services, although their involvement is limited [27].

Underutilization of available services in the country was consistently reported [15, 16, 24, 27, 30]. The main reason for pastoralists not using health services was long travel distance to health care facilities, reported by more than 60% [16, 25, 27]. At the national level, distance as a reason for non-use was only stated by 9% [30]. At the national level, 41% of the respondents reported cost-related as a reason for non-use, whereas for pastoralists, only 8–34% reported cost as a deterrent [26, 27].

#### Pastoralists’ knowledge and awareness on TB control services

Overall, the studies reported that pastoralists were well aware of TB as a disease, despite their low literacy level. Pastoralists also had good knowledge on transmission and preventive mechanisms for TB. The main sources of information reported were media (primarily radio), relatives and health professionals. However, major misconceptions were also reported. A striking misconception was that some pastoralists consider a persistent cough to be an ordinary event unless it is also accompanied by blood [13, 24].

The common treatment options were also familiar to the participants. When asked which treatment option was preferred, most respondents favoured conventional care [15, 16], yet a considerable number of the respondents utilized traditional medicines before they sought biomedical care [13, 24].

#### Challenges of TB control in pastoral settings

The reviewed articles reported challenges, which hamper the effectiveness of TB control in some Ethiopian pastoralists. Table 1 lists challenges according to the related effectiveness parameters.

**Table 1:** TB control challenges

a) Adequacy	
- Limited experience [26], lack of supervision [28] and in-service training [27] for the staff on duty.	
- Poor staffing quality [28]	
- Incomplete record keeping [28]	
- Insufficient level of decentralisation [18, 24]	
- Inadequate health infrastructure [24]	
- Absence of professionals during working hours [28]	
- Long waiting time [26, 28]	
b) Adherence	
- High default rate due to pastoral mobility contrasted with static health facilities [28]	
c) Availability	
- Shortage of trained staff [18, 24]	
- Intermittent shortages/stock outs of drugs and laboratory supplies [26, 28]	
d) Targeting accuracy	
- Inadequate Acid-Fast Bacilli (AFB) microscopy follow-up [28]	
- Very low detection rate [18, 32] and low sensitivity of diagnosis (microscopy) [18]	
e) Acceptability	
- Lack of trust of health extension workers [27]	
- Preference of traditional medicinal practices [24, 26]	
- Perceived symptoms as self-limiting [27]	
f) Accessibility	
- Services are only available in major towns and villages along main roads [24]	
- Inaccessibility of service [27], including lack of transport infrastructure [24]	
- Long distance to travel for service [16, 23, 24, 27]	
g) Affordability	
- Higher expenses incurred during the first two months of treatment [24]	
- Cost of transportation and housing during the intensive phase [16, 24, 26, 27]	

#### Equity disparities affecting pastoralists

TB control activities in pastoralist regions of Ethiopia were far fewer than those observed elsewhere in the country. The first national TB prevalence survey in 2010/2011 reported that pastoralists had the highest prevalence, at 291 (range: 126–456) per 100 000 population throughout the country [4]. The prevailing inequity and how pastoral communities were particularly affected was acknowledged in the Health Sector Development Programme (HSDP) IV (2010/2011–2014/2015) document [33]. One study reported disproportionate inaccessibility of health service for pastoralists [27]. Health service providers and regional policy planners stated that pastoralists would not fulfil the requirements for bringing service to their proximity due to low pastoralist population density, unpredictable mobility and high staff turnover in remote areas [24].

**Fig. 2:**
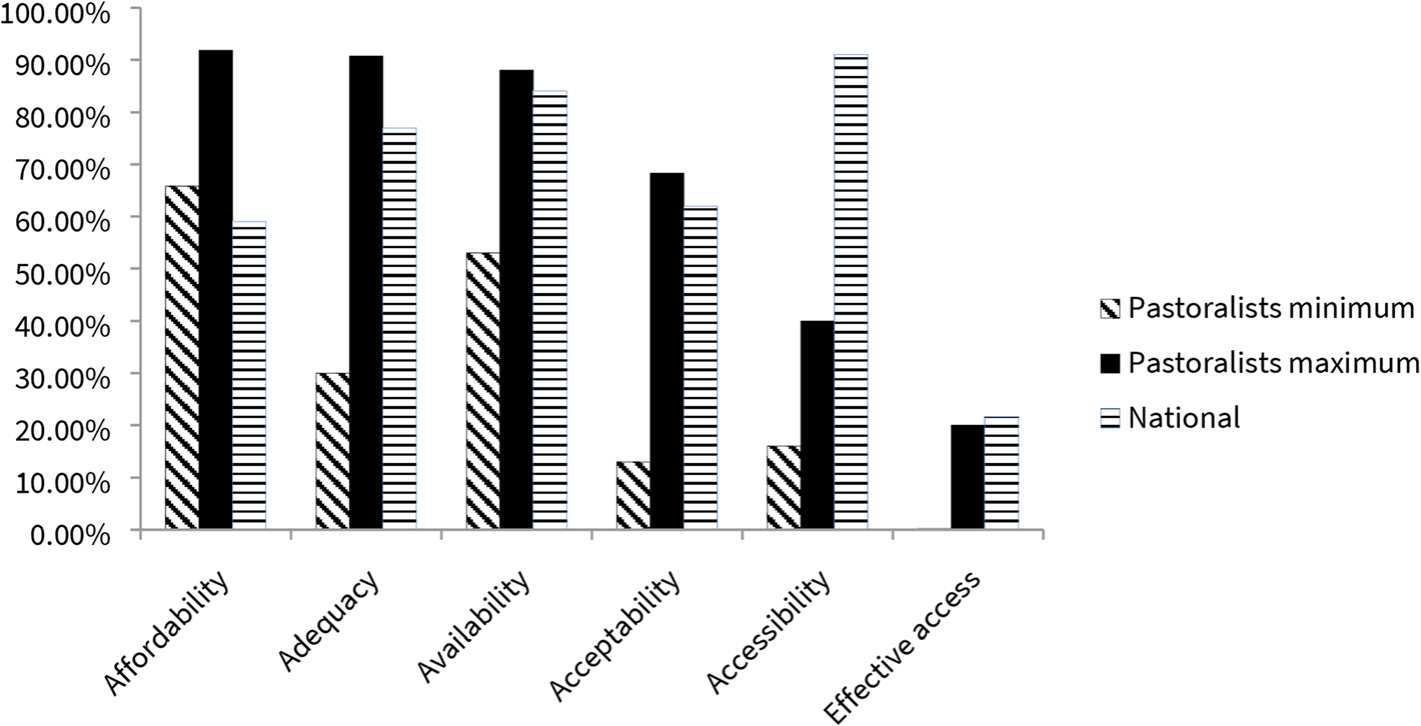
Contribution of access parameters to the effectiveness of tuberculosis services in Ethiopia. Values are based on Table 2 with results rounded up to the nearest integer

When addressing inequities, thoughtful considerations of the social, economic, cultural, biological and environmental determinants of health are crucial [34]. Hence, assessing equity-effectiveness in TB requires evaluating both quality of care and access to services. Figure 2 shows advantages of different parameters of equity-effectiveness.

Using the method described by Zinsstag and colleagues [6] with the data shown in the analysis column of Table 2, the estimated final community-effectiveness was 6.3% nationally and ranged from 0.01 to 1.2% for pastoralists.

**Table 2:** Assessment of effectiveness parameters

Effectiveness parameter	Description	Quantitative assessment	Qualitative assessment	Analysis
Efficacy	An estimate of the ability of anti-TB drugs to cure the disease	- Results from clinical trials	- A biological feature of the drugs	
Targeting accuracy	How well were the cases identified	- A 10-year trend analysis study in SRS reported average case detection rate (CDR) of 19.1% [18] - Another 10-year trend study in Afar region showed 34% CDR - Calculated CDR of 69.1%, from 2017 WHO Global TB Report [2]		In pastoralist areas 19.1–34.0% of TB cases were detected, while at national level 69.1% of cases were detected
Availability	Service meets patient’s needs	- As of 2016, 53% of the available facilities in SRS had TB microscopy and 88% had all first-line anti-TB drugs [35] - A study conducted in Afar region reported 28.7% of participants were dissatisfied with service provision [28] - 84% of health facilities in Ethiopia had all first-line anti-TB drugs [35]	- Shortage of trained staff and intermittent stock outs of drugs and laboratory supplies - Available services are concentrated in major towns	For effectiveness estimation: at national level 84% of the required drugs were available while 53–88% of the required services were available in pastoralist areas.
Accessibility	Health service delivery sites are accessible to the community without significant physical or social barriers	- Travel distance was too far for 60% of participants [27] - More than 84% of pastoralists in SRS lacked access to TB care [24] - At national level, 9% of the population did not use services because health facilities were too far away	- Lack of transport infrastructure (roads), long travel distances to health facilities	16–40% of health facilities were accessible to pastoralists, while national level accessibility was reported as 91%
Affordability	Willingness and ability to pay cost of TB service	- In SRS 8.2% delayed treatment due to lack of money [27] - In Bale zone of Oromia 34.2% of pastoralists delayed treatment due to lack of money [26] - At national level 41% reported inability to pay for the service [30]	- The economic burden of TB was the most important concern for pastoralists in SRS [24]	65.8–91.8% of pastoralists could afford the cost of the service, while at national level 41% could not afford it
Adequacy	The TB service organisation meets the community’s expectations	- 9.3% of participants in a pastoralist zone of Oromia region delayed treatment because of long waiting time [26] - Dissatisfaction rate in waiting time (70%) and in open hours (63.6%) was reported in Afar region [28] - 77% of the outpatient health provision was from public health services [23]	- Limited supervision and in-service training of staff, poor staffing quality - Insufficient decentralisation of DOTs and inadequate health infrastructure	30.0–97.7% of pastoralists were satisfied with the service, while at national 77% of the population utilised public health services
Acceptability	Provider’s characteristics match those of the community	- 87% of pastoralists in SRS preferred traditional medicine [23] - 31.7% of participants reported their symptoms as self-resolving [27] - Health care seeking at national level was 62% [30]	- Lack of trust in health extension workers - Preference for traditional medicine and other informal health services	13.0–68.3% of patients felt comfor\ with the conventional service providers. In contrast, at national level 62% sought health care
Compliance	How well provider initiates correct procedure for intervention- How well health care providers conform to standards of TB treatment	- Despite availability of services, only two of six (33.3%) health facilities studied in Afar region followed strict DOTs during the intensive phase [28] - 44% of Ethiopian health facilities had diagnostic and treatment guidelines	- Absence of professionals during working hours	33.3% of health facilities in pastoral area followed strict DOTs, while at national level 44% of health facilities followed standard guidelines
Adherence	How well recipient follows medical advice given	- In SRS 4.2% of patients enrolled for TB treatment defaulted over the period 2003–2012 [18]. Default rate was 2.9% in Afar region [19]	- High default rate due to pastoralist mobility versus stationary health facility	95.8% of patients adhered to treatment in pastoral settings

## Discussion

The data stemming from our systematic review, supplemented with a meta-ethnographic approach, triangulates results across the reviewed studies to present a line of argument describing the contextual status of TB control services in Ethiopian pastoralists.

### Pastoralism and TB care in Ethiopia

Pastoralism has long been practiced throughout the world and proven to be a sustainable livelihood option [34]. In Ethiopia, pastoralists are either nomads or agro-pastoralists. Our findings show that TB is familiar to pastoralists, but the biomedical concept of the causative agent is unknown. This observation might explain why pastoralists prefer traditional medical practices.

### Pastoralists’ access to TB service

Health seeking begins when pastoralists identify a place to get help and secure the means to travel and remain during the course of treatment. One of the reviewed studies [21] described a pilot intervention where patients were provided culturally acceptable food, shelter and other utilities during the treatment course and reported a treatment success rate of 92%. This approach was implemented in Afar Region in 2001 [35] and in Kenya in 1987 [36]. However, the cost of the intervention was high and the scale up and sustainability of such a strategy does not seem feasible. Similar interventions have been reported in northern Ethiopia, with some variations in approach [37, 38].

### Equity-effectiveness in TB care

The Health Sector Transformation Plan (HSTP) of the Ethiopian government emphasises the need for equitable health services for all. Other policies previously included pastoralists, such as the “Pastoralist Health Service” of HSDP IV and the “Mobile Health and Nutrition Team” of HSDP II [33]. While these plans indicate governmental commitment, disproportionate inaccessibility of health service for pastoralists persist [27]. Although health service providers related these discrepancies to pastoralists’ low population density, unpredictable mobility and high staff turn-over in remote areas [24], we argue that the most relevant reason for the policy/practice discrepancy is that the plans were not tailored to the local context because pastoralists, whose experiences, disease perceptions and suggestions are essential for community ownership, were not involved in the planning phase.

Our findings indicate that inaccessibility and lack of acceptability to TB care are the key bottlenecks to pastoralist TB service provision. Targeting these two parameters will contribute to enhance effectiveness of an intervention. Specifically, incorporating traditional medical practitioners and other locally important stakeholders, such as traditional birth attendants, religious leaders and community elders, into the TB care continuum might enhance effectiveness of TB control. Further expansion of services in to closer proximity of communities, improvements in transport infrastructure, and provision of mobile services to match pastoralist lifestyle will substantially improve intervention impact.

### Limitations of the study

This review is limited by the quality of the included studies. To assure reasonable quality, we extracted information from included studies by strictly following a systematic procedure. In calculating the access and effectiveness parameters, we applied the conditional probability product of the parameters by assuming that parameters are independent of each other. However, this may not always be accurate, so the model should be reviewed against other effectiveness models as these become available.

## Conclusions

In the reviewed studies, we found that TB control in Ethiopia did not align well with the pastoralist lifestyle, and most authors expressed the need to fit TB service provision to the pastoralists’ lifestyle. Interventions using modified treatment strategies reported treatment outcomes similar to that of DOTS as implemented in stationary health facilities [21, 22]. This may be because these modified interventions did not consider mobility of pastoralists and their connection to livestock.

For Ethiopian pastoralists, we recommend an intervention which incorporates community members with established backgrounds of trust and experience in community services, such as traditional healers, religious teachers, traditional birth attendants and other stakeholders, into public health care. There is a need to provide training in basic principles and practices of One Health. These principles should be focused on joint climate-resilient human and animal health and social resilience. Collaborating with community members and empowering them to execute health service provision activities will enhance the effectiveness of health service in pastoral settings.

## Additional files

Additional file 1: Multilingual abstracts in the five official working languages of the United Nations. (PDF 250 kb)

Additional file 2: Characteristics of included studies. (DOCX 40 kb)

## Data Availability

All data generated or analysed during this study are included in this published article and its Additional files.

## Abbreviations

AJOL: Africa Journals Online
CABI: Centre for Agriculture and Biosciences International
CDR: Case detection rate
DOTS: Directly observed treatment, short-course
EPTB: Extra-pulmonary tuberculosis
HEWs: Health extension workers
HIV/AIDS: Human immune deficiency virus/ acquired immune deficiency syndrome
HSDP: Health Sector Development Programme
HSTP: Health Sector Transformation Plan
JOHI: Jigjiga One-Health Initiative
MDR TB: Multidrug resistant tuberculosis
MeSH: Medical Subject Heading
MTC: *Mycobacterium tuberculosis* complex
NGOs: Non-Governmental Organisations
OOP: Out-of-pocket
PICO: Population (participants), Intervention, Comparator and Outcomes
PRISMA: Preferred Reporting Items for Systematic Reviews and Meta-analysis
PTB: Pulmonary tuberculosis
RCT: Randomized controlled trial
SAT: Self-administred treatment
SRS: Somali Regional State
TB: Tuberculosis
USD: United States Dollar
WHO: World Health Organization
