# Supplementary material for: The impact of pastoralist mobility on tuberculosis control in Ethiopia: a systematic review and meta-synthesis
Source: Infect Dis Poverty. 2019 Sep 2;8:73. doi: 10.1186/s40249-019-0583-z (PMC6717972; doi:10.1186/s40249-019-0583-z)
Supplement: Supplementary file 1 — Multilingual abstracts in the five official working languages of the United Nations. (PDF 250 kb) [file 40249_2019_583_MOESM1_ESM.pdf]

Translation of the abstract into the five official working languages of the United Nations

المراجعة المنهجية والتوليف التلوي للسيطرة على مرض السل في إثيوبيا مع التركيز بشكل خاص على الرعاة: التعديلات على ((الدورة العلاجية القصيرة الأجل بالملاحظة المباشرة)) للرعاة المتنقلين

فيصل نوح، ليزا كرمب، عبد الوهاب حاشي، ريا تشوب، إستير شيلينغ، كلاوس ريثر، جان هاتيندورف، سعيد م. علي، بريجيت أوبريست، يورغ أوتزينجر، جاكوب زينستاج

الخلاصة

الخلفية: تعد ((الدورة العلاجية القصيرة الأجل بالملاحظة المباشرة (DOTS)) على المستوى العالمي بمثابة الإستراتيجية الرئيسية للسيطرة على مرض السل. الإمكانية المتوخاة من ((الدورة العلاجية القصيرة الأجل بالملاحظة المباشرة)) في السيطرة على السل وتحسين الصحة هو أمر معترف به على نطاق واسع. وعلى وجه الخصوص، لقد ثبت أن التعديلات الخاصة بسياق ((الدورة العلاجية القصيرة الأجل بالملاحظة المباشرة)) لها آثار وشيكة في مكافحة السل. على الرغم من أن البرنامج الوطني لمكافحة السل في اثيوبيا يوفر آفاقاً وأن الدولة تضع خططاً للقضاء على السل، فإن التوزيع غير المتساوي الشامل عبر التدرجات في أنماط/أساليب الحياة لا يزال يمثل تحدياً. الجدير بالذكر أن المجتمعات الرعوية المتنقلة في البلاد تُترك بلا تغطية بشكل غير متناسب. وفي هذا الصدد، كان الهدف من هذه الدراسة هو إنشاء قاعدة أدلة من المواد المنشورة لتوجيه استراتيجية مكافحة السل للمجتمعات الرعوية المتنقلة في إثيوبيا.

النص الرئيسي: اتبعنا إرشادات ((بنود التقارير المفضّلة للمُراجعات المنهجيّة والتحليل التلوي (PRISMA)) من أجل مراجعة المقالات بشكل منهجي في سبع قواعد بيانات إلكترونية هي: ، African Journal Online (AJOL) ، EMBASE Direct، Cochrane (المركز الدولي للزراعة والعلوم البيولوجية) MEDLINE/Pubmed،Google Scholar ، CABI Library، Web of Science. تم تضمين المواد المنشورة قبل 31 ديسمبر/كانون الأول 2018.

قمنا بفحص 692 عنوان وتم اختيار 19 منها تفي بمعايير الادراج. حددنا ستة موضوعات/محاور في عملنا هي: (1) الرعي في إثيوبيا (2) لمحة عن سبل معيشة الرعاة (3) استخدام خدمات الرعاة (4) معرفة ووعي الرعاة بخدمات مكافحة السل (5) تحديات مكافحة السل في البيئات الرعوية (6) التفاوتات/الانصافات التي تؤثر على الرعاة. يحدد تفسيرنا النتائج في جميع الدراسات المشمولة ويظهر أن أنشطة مكافحة السل في المناطق الرعوية في إثيوبيا أقل بكثير عن تلك المرصودة في أماكن أخرى من البلاد.

استنتاج مقتضب: توضح هذه المراجعة أن مكافحة السل في إثيوبيا لم تتماشى بشكل جيد مع نمط الحياة الرعوية. عدم القدرة على الوصول (والعجز عن الوصول) إلى رعاية مرضى السل هما من العقبات الرئيسية التي تعترض توفير خدمة مرضى السل الرعوية. استهداف هاذين العاملين يسهم في فعالية التدخل.

Translated from English version into Arabic by Ammar Alhosain, Revised by Ahmed Ibrahim, through

TRANSLATORS WITHOUT BORDERS

关注埃塞俄比亚牧民结核病控制的系统综述和 **meta** 分析：**DOTs** 对流动性牧民的适应性

Faisal Nooh, Lisa Crump, Abdiwahab Hashi, Rea Tschopp, Esther Schelling, Klaus Reither, Jan Hattendorf, Said M. Ali, Brigit Obrist, Jürg Utzinger and Jakob Zinsstag

摘要

引言：直接督导短程化疗（DOTs）是全球控制结核病的主要策略。DOTs 对于控制结核病和改善健康的效果已得到广泛认可，尤其是特定环境下采用 DOTs。在埃塞俄比亚，尽管国家结核病控制规划的前景良好，国家也制定了消灭结核病的计划，但也面临着一些挑战，如人民生活方式的差异。值得注意的是，该国流动牧民群体并没有获得很好的防治措施。鉴于此，本研究旨在从已发表的文献中获得证据从而指导埃塞俄比亚流动牧民的结核病控制策略。
正文：根据 PRISMA 指南，我们从 7 个电子数据库中筛选进行系统性综述，分别是 EMBASE、非洲在线期刊(AJOL)、MEDLINE/Pubmed、谷歌学术、CABI(国际农业和生物科学中心)Direct、Cochrane 图书馆和 Web of Science。2018 年 12 月 31 日前发表的文献都被纳入其中。我们对 692 个项目进行了筛选，其中 19 个项目符合纳入标准。我们在文献中制定了 6 个主题:(1)埃塞俄比亚的游牧主义；(2)牧民生产资料；(3)牧民获得的服务；(4)牧民对结核病控制措施的了解和认识；(5)牧区结核病控制面临的挑战；(6)卫生服务差距对牧民的影响。综合所纳入的研究结果表明，在埃塞俄比亚畜牧地区采取的结核病控制措施远远少于该国其它地区。
结论：经综述表明，埃塞俄比亚的结核病控制措施并不适于牧民的生活方式。难以获得服务和缺乏合适的护理措施是牧民结核病服务的主要障碍。针对以上两点制定相关方案将有助于提高干预的有效性。

Translated from English version into Chinese by Peng Song, edited by Jin Chen

CDC CHINESE CENTER FOR DISEASE CONTROL AND PREVENTION
NATIONAL INSTITUTE OF PARASITIC DISEASES

Revue systématique et méta-synthèse de la lutte antituberculeuse en Éthiopie avec un accent particulier sur les pasteurs : adaptations des DOT aux pasteurs mobiles

Faisal Nooh, Lisa Crump, Abdiwahab Hashi, Rea Tschopp, Esther Schelling, Klaus Reither, Jan Hattendorf, Said M. Ali, Brigit Obrist, Jürg Utzinger et Jakob Zinsstag

Résumé

Contexte : Le traitement de brève durée sous surveillance directe (DOTS) constitue globalement la principale stratégie de lutte contre la tuberculose (TB). Le potentiel de la stratégie DOTS dans la lutte contre la TB et l'amélioration de la santé est largement reconnu. En particulier, les adaptations de la stratégie DOTS spécifiques au contexte ont des implications imminentes dans la lutte contre la TB. En Éthiopie, bien que le programme national de lutte antituberculeuse offre des perspectives, et bien que le pays élabore des plans pour éliminer la TB, la répartition inégale générale entre les différents modes de vie reste un défi. Notamment, les communautés pastorales mobiles du pays sont laissées sans couverture, et ceci de manière disproportionnée. À cet égard, l'objectif de cette étude était de générer une base de preuves issues d’études publiées afin de guider une stratégie de lutte contre la TB pour les communautés pastorales mobiles en Éthiopie.
Texte principal : Nous avons suivi les lignes directrices PRISMA pour examiner systématiquement les articles dans sept bases de données électroniques : EMBASE, African Journal Online (AJOL), MEDLINE/Pubmed, Google scholar, CABI (The Centre for Agriculture and Bioscience International) Direct, Cochrane Library et Web of Science. Les études publiées avant le 31 décembre 2018 étaient incluses.

Nous avons examiné 692 articles et en avons sélectionné 19 qui répondaient aux critères d'inclusion. Nous avons identifié six thèmes dans les études : (i) le pastoralisme en Éthiopie ; (ii) le profil des moyens d'existence des pasteurs ; (iii) l'utilisation des services des pasteurs ; (iv) les connaissances et la sensibilisation des pasteurs sur les services de lutte contre la TB ; (v) les défis de la lutte antituberculeuse en milieu pastoral ; et (vi) les inégalités affectant les pasteurs. Notre interprétation regroupe les résultats de toutes les études incluses et montre que les activités de lutte contre la TB dans les régions pastorales de l'Éthiopie sont beaucoup moins nombreuses que celles observées ailleurs dans le pays.
Brève conclusion : Cette revue montre que la lutte antituberculeuse en Éthiopie ne s'alignait pas correctement avec le style de vie des pasteurs. L'inaccessibilité et le manque d'acceptabilité des soins antituberculeux sont les principaux obstacles à la prestation de services de lutte contre la TB pour les pasteurs. Le ciblage de ces deux paramètres contribuera à l'efficacité d'une intervention.

Translated from English version into French by Emilie Rigault Fourcadier, Revised by Fabrice M'Vondo, through

TRANSLATORS
WITHOUT BORDERS

Систематический обзор и метасинтез распространенности туберкулеза в Эфиопии с особым акцентом на работников в сфере животноводства: изменения в лечении под непосредственным контролем, коротким курсом (DOTS) для кочевых скотоводов

Faisal Nooh, Lisa Crump, Abdiwahab Hashi, Rea Tschopp, Esther Schelling, Klaus Reither, Jan Hattendorf, Said M. Ali, Brigit Obrist, Jürg Utzinger and Jakob Zinsstag

Аннотация

История вопроса: В мировом масштабе лечение под непосредственным контролем, коротким курсом (DOTS) является основной стратегией борьбы с туберкулезом (ТБ). Широко признан потенциал лечения под непосредственным контролем, коротким курсом (DOTS) в борьбе с ТБ и в улучшении здоровья. В частности, было показано, что адаптация лечения под непосредственным контролем, коротким курсом (DOTS) к конкретным условиям имеет непосредственное значение в борьбе с ТБ. В Эфиопии, несмотря на то, что национальная программа борьбы с ТБ предоставляет возможности, и в стране разрабатываются планы по ликвидации ТБ, проблемой по-прежнему остается всеобъемлющее неравномерное распределение ресурсов во всех сферах жизни. В частности, сообщества кочевых скотоводов страны остаются без внимания. В связи с этим целью данного исследования было получение доказательной базы на основе опубликованной литературы для предоставления руководства по стратегии борьбы с ТБ для сообщества кочевых скотоводов в Эфиопии.

Основной текст: Мы следовали руководствам PRISMA при систематическом обзоре статей из семи электронных баз данных: EMBASE, African Journal Online (AJOL), MEDLINE/Pubmed, Google scholar, CABI (The Centre for Agriculture and Bioscience International) Direct, Cochrane Library и Web of Science. Были включены литературные источники, опубликованные до 31 декабря 2018 г.

Мы провели скрининговое исследование 692 объектов и выбрали 19, которые соответствовали критериям. В литературных источниках было выделено шесть тем: 1) животноводство в Эфиопии; 2) средства к существованию скотоводов; 3) использование услуг скотоводов; 4) знания и осведомленность скотоводов о службах по борьбе с ТБ; 5) проблемы при борьбе с ТБ в пастбищных условиях и 6) диспропорция в справедливости, затрагивающая скотоводов. Результаты по всем включенным исследованиям обобщаются с точки зрения нашего понимания и свидетельствуют о том, что меры по борьбе с ТБ в скотоводческих регионах Эфиопии внедряются намного реже, чем где-либо еще в стране.

Краткие выводы: Данный обзор показывет, что меры по борьбе с ТБ в Эфиопии надлежащим образом не сответствуют образу жизни скотоводов. Недоступность и непринятие противотуберкулезной помощи являются основными препятствиями для предоставления скотоводам медицинских услуг по борьбе с ТБ. Ориентация на эти два аспекта будет способствовать эффективности мероприятий.

Translated from English version into Russian by Veronika Demeshchyk, Revised by Anna Kukharchuk, through

TRANSLATORS
WITHOUT BORDERS

El análisis sistemático y la metasíntesis del control de la tuberculosis en Etiopía con especial énfasis en los pastores: adaptaciones de la DOTS para pastores nómadas

Faisal Nooh, Lisa Crump, Abdiwahab Hashi, Rea Tschopp, Esther Schelling, Klaus Reither, Jan Hattendorf, Said M. Ali, Brigit Obrist, Jürg Utzinger y Jakob Zinsstag

Resumen

Antecedentes: la terapia observada directamente de corta duración (DOTS, por sus siglas en inglés), a nivel mundial, es la principal estrategia para controlar la tuberculosis (TB). El potencial de la DOTS para controlar la tuberculosis y mejorar la salud es ampliamente reconocido. En particular, se ha demostrado que las adaptaciones específicas de la DOTS según el contexto tienen implicaciones inminentes en la lucha contra la tuberculosis. En Etiopía, aunque el programa nacional de control de la tuberculosis ofrece perspectivas y el país establece planes para eliminar la tuberculosis, la distribución desigual general entre los gradientes de estilo de vida sigue siendo un desafío. Cabe destacar que las comunidades de pastores nómadas en el país quedan desproporcionadamente descubiertas. En este sentido, el objetivo de este estudio fue generar una

base de pruebas a partir de la literatura publicada, a fin de guiar una estrategia de control de la TB para las comunidades de pastores nómadas en Etiopía.

Texto principal: seguimos las directrices de PRISMA para revisar sistemáticamente artículos en siete bases de datos electrónicas: EMBASE, African Journals Online (AJOL), MEDLINE / Pubmed, Google Scholar, CABI (Centro de Agricultura y Biociencia Internacional) Direct, Cochrane Library y Web of Science. Se incluyó la literatura publicada antes del 31 de diciembre de 2018.

Examinamos 692 artículos y seleccionamos 19 que cumplían los criterios de inclusión. Identificamos seis temas en la literatura: i) pastoreo en Etiopía, (ii) perfil de subsistencia de los pastores, iii) la utilización de servicios de los pastores, iv) el conocimiento y la sensibilización de los pastores sobre los servicios de control de la tuberculosis, v) los desafíos del control de la tuberculosis en entornos pastorales, y vi) las disparidades de equidad que afectan a los pastores. Nuestra interpretación triangula los resultados de todos los estudios incluidos y muestra que las actividades de control de la tuberculosis en las regiones pastoriles de Etiopía son mucho menos de las observadas en otras partes del país.

Conclusión breve: este análisis muestra que el control de la tuberculosis en Etiopía no se ajustó bien al estilo de vida de los pastores. La inaccesibilidad y la falta de aceptabilidad del cuidado de la TB son los principales obstáculos para la prestación de servicios de TB para los pastores. Abordar estos dos parámetros contribuirá a la eficacia de una intervención.

Translated from English version into Spanish by María Luz, Revised by Mayra León, through

TRANSLATORS
WITHOUT BORDERS
