# Supplementary material for: The impact of pastoralist mobility on tuberculosis control in Ethiopia: a systematic review and meta-synthesis
Source: Infect Dis Poverty. 2019 Sep 2;8:73. doi: 10.1186/s40249-019-0583-z (PMC6717972; doi:10.1186/s40249-019-0583-z)
Supplement: Supplementary file 2 — Characteristics of included studies. (DOCX 40 kb) [file 40249_2019_583_MOESM2_ESM.docx]

Supplement file A. Characteristics of included studies

|  | Authors | Objective | Study characteristics | | | Participant characteristics | | Major findings |
| --- | --- | --- | --- | --- | --- | --- | --- | --- |
|  |  |  | Duration | Data collection tools | Design | Number | Type |  |
| 1 | Getnet et al., 2017 (27) | To examine the involvement of health extension workers (HEWs) in TB case notification in Somali region | Jun - Nov 2014 | Questionnaire and interview | Cross-sectional survey and in-depth interview | 385 | TB patients and HEWs | Proportion of pulmonary tuberculosis (PTB) patients referred by HEWs (20.3% [16.6- 24.5%]), by health care workers (52.6%), by family member (13.4%), by neighbours/friends (2.4%), by self (11.3%) |
| 2 | Gele et al., 2010 (24) | To investigate diagnostic delay among pastoralists and associated factors in Somali region | Jun - Sept 2007 | Participatory rural appraisal (PRA) and informal interview | Qualitative study | 19 | Community members | Lack of access to formal health services as well as traditional beliefs leading to self-treatment; nomadism and agro-pastoralism as the main lifestyle in the study area; catastrophic out of pocket expenditure |
| 3 | Gele et al., 2009 (23) | To measure duration of delay among pastoralist TB patients in Somali region | Jun– Sept 2007 | Questionnaire | Health facility based cross-sectional | 226 | TB patients | Median patient delay was found to be 60 days, median health care provider's delay was 6 days, median total delay was 70 days |
| 4 | Belay et al., 2012 (25) | To measure the duration of diagnostic and treatment delays among pastoralists in Afar region | Sep 2009 to March 2010 | Questionnaire | Health facility based cross-sectional study | 216 | TB patients | Median patient delay was found to be 20 days, median health care provider's delay was 33.5 days, median total delay was 70.5 days |
| 5 | Hussen et al., 2012 (26) | To investigate factors associated with treatment delay among pastoralists in Bale zone of Oromia region | Feb – Mar 2011 | Questionnaire | Health facility based cross-sectional study | 129 | TB patients | Median patient delay was found to be 63 days, median health care provider's delay was 34 days, median total delay was 97 days |
| 6 | Girma et al, 2010 (28) | To evaluate TB care quality in Afar region | Feb – Mar 2007 | Record reviews, interviews and observations | Health facility based cross-sectional study | 276 | TB patients & service providers | Fairly good supply of drugs and laboratory materials; poor staffing qualities; weak supervision of workers; 63.5% of patients were not satisfied with the working hours; 70% of patients complained of long waiting time |
| 7 | Khogalia et al., 2014 (22) | To assess treatment outcomes of a modified self-administered treatment strategy for pastoralists with TB in Somali region | May 2010 to Mar 2012 | Records review | Cohort study | 390 | TB patients | Overall treatment success rate was 81.2% (317/390); the rates of death, loss-to-follow up and treatment failure were 6.7% (26/390), 9.2% (36/390) and 0.3% (1/390) |
| 8 | Tayler-Smith, et al., 2011 (21) | To describe and evaluate an implemented TB village strategy for pastoralists in Somali region | Sep 2006 to Oct 2008 | Records review | Health facility based cross-sectional study | 340 | Patients | 54% smear positive TB, 49% smear negative PTB, 19% extra-pulmonary TB (EPTB); 11% unrecorded outcome, 45% failure, 91% treatment success rate, 3% default, 4% death |
| 9 | Legesse et al., 2010 (13) | To explore the knowledge and perceptions of pastoralists for TB in Afar region | Mar to May 2009 | Questionnaire and focus group discussion | Community-based cross-sectional study | 818 | Community members | 95.6% knew TB as a disease; only 0.3% mentioned bacteria as the causative agent. Causes of TB was reported as cold air (45.9%), starvation (38%), dust (21.8%) or smoking/chewing Khat (Catha edulis) (16.4%); 74.3% knew the major signs and symptoms; 94.2% knew that TB is curable |
| 10 | Melaku et al., 2013 (14) | To assess knowledge, attitudes and practices regarding TB among pastoralists in Somali region | Feb to May 2011 | Questionnaire | Community-based cross-sectional study design | 821 | Community members | 92.8% knew TB as a disease; 10.1% mentioned bacteria as the causative agent. More than 30% knew the major signs and symptoms; 41.3% knew the transmission and preventive measures; 98.3% reported that TB could be cured, of which 93.3% believed with modern drugs |
| 11 | Tolossa et al., 2014 (16) | To examine communities’ knowledge, attitude and practices towards TB in Somali region | Jan to May 2013 | Questionnaire | Community-based cross-sectional study design | 410 | Community members | 94.9% knew TB as a disease; 22.9% identified bacteria as the cause. 80% knew transmission methods, 79.3% knew that TB is preventable. 72.4% knew persistent cough as the main symptom and 68.1% preferred modern drugs. 71.0% reported to prefer health facilities to traditional medicine |
| 12 | Sima et al., 2017 (15) | To compare knowledge, attitude and practice among mobile pastoralists and sedentary communities in Oromia region | Sep 2014 to Jan 2015 | Questionnaire and focus group discussion | Community-based cross-sectional study design | 610 | Community members | Identification of bacteria as the cause of TB was 63.9% in pastoralists and 81% in the sedentary group. 95.8% of pastoralists indicated PTB is preventable while in the sedentary group 99.6% reported |
| 13 | Nigatu and Abraha, 2010 (17) | To examine the epidemiological trends of TB in Ethiopia over the period 2000-2009 | 2000-2009 | Report review | Health facility report based time series study |  |  | Annual increment rate in incidence was 5 new TB cases per 100000 populations per year over the ten year period. Urban agro-ecological zones were more affected by the disease throughout the ten-year period; extra-pulmonary rate and smear-negativity showed a modest increment during the study period; males were disproportionately affected by TB |
| 14 | Woldeyohannes et al., 2015 (18) | To evaluate the impact of DOTs strategy on new TB case finding and treatment outcomes in Somali region over 10-year period | 2003-2012 | Records review | Health facility report based time series study | 31,198 |  | Smear positive TB (40%), smear negative TB (33.8%), EPTB (26.2%); case detection rate (19.1%), treatment success rate 85.5% (73.1-90.8%), default 4.2% (2.3-4.7%), failure 0.8% (0.0-1.3%) |
| 15 | Tafess et al., 2016 (19) | To evaluate the impact of DOTs strategy on new TB case finding and treatment outcomes in Afar region over ten year period | 2003-2012 | Records review | Health facility report based time series study | 34,894 |  | 2.7% failure, 33.2% smear positive TB, 39.7% smear negative TB, 28.2% EPTB, 32% case detection rate, 86.2% treatment success rate, 2.9% default |
| 16 | Ethiopian Public Health Institute (20) | To assess the trends in hospital admissions, outpatient consultations and mortalities related to HIV/AIDS, TB and malaria in Ethiopia | 2012-2016 | Facility-based register, HDSS data and mortality surveillance programme data review | Time series study | 3,650 from HDSS and 9,905 from AAMP |  | Consultation due to TB decreased from 5% to 1.5% over the period 2012-2016; TB contributed 8.8% to the total death; Proportionate mortality ratio of TB was 6% |
| 17 | Environmental Protection & Energy & Mines Resources Development Agency (29) | To assess the implications of climate change on pastoral and agro-pastoral livelihoods in Somali region. |  | Interview | Qualitative study | 100 | Local authorities and community members | Recommended involvements for adapting to climate change |
| 18 | Federal Democratic Republic of Ethiopia Ministry of Health (30) | To generate sound empirical evidence on household health services utilization and spending on health in Ethiopia | Dec 2012 to Jan 2013 | Questionnaire | Cross-sectional survey | 10,060 | Household | In 2012 more than 90% of households reported good or very good health status; incidence of illness was reported by 12% of the population; health care service were sought by 62%; reasons for not seeking health care were lack of money (41%), not recognizing the severity of illness (25%), self-care (15%), long distance to travel (9%), poor quality of health care (3%); non-communicable diseases contributed 5% of consultations, 7% of admissions, 13% of deaths, outpatient health care provision was 77% by public, 20% private, 2% traditional healers, 1% NGOs; total annual OOP health expenditure was USD 590 Mio, per capita = USD 7.49 |
| 19 | Zemedu et al., 2015 (31) | To assess TB service availability in Ethiopian health facilities |  | Record review and questionnaire | Cross-sectional | 1,327 | Health facilities and health workers | 29% of the health posts and 69% of the other health facilities in the country provide all required TB services; 60% of them have trained staff; 59% of the health facilities other than health posts have Acid-fast Bacilli microscopy and 44% have guidelines |
